# Behind the Swelling: Primary Mediastinal Large B-cell Lymphoma Masquerading as an Infection

**DOI:** 10.7759/cureus.101839

**Published:** 2026-01-19

**Authors:** Muhammad Ali Lak, Dina Abdelhamid, Viren S Sehgal, Arianna Falletta, Natalia Skrodzka, Merjona Saliaj

**Affiliations:** 1 Medicine, Icahn School of Medicine at Mount Sinai, Queens Hospital Center, New York City, USA; 2 Internal Medicine, City University of New York School of Medicine, New York, USA; 3 Internal Medicine, New York Institute of Technology College of Osteopathic Medicine (NYITCOM), Westbury, USA

**Keywords:** actinomyces, anchoring bias, leukemoid reaction, mediastinal mass, primary mediastinal large b-cell lymphoma (pmbcl), superior vena cava syndrome

## Abstract

We present the case of a 26-year-old female patient with a history of asthma who initially presented with symptoms suggestive of a common infection. Her constellation of facial swelling, productive cough, and fatigue, coupled with imaging findings, first led to a diagnosis of right upper lobe pneumonia and suspected cellulitis. Despite appropriate antibiotic therapy, her symptoms persisted, and her clinical picture became more complex with the identification of *Actinomyces* in her sputum, shifting the differential diagnosis toward a more indolent cervicofacial infection. However, a progressively worsening and profound leukocytosis, along with a lack of clinical improvement, prompted further investigation. This ultimately revealed a large mediastinal mass, and subsequent tissue analysis confirmed a diagnosis of primary mediastinal large B-cell lymphoma. This case highlights a critical diagnostic challenge, illustrating how a rare and aggressive malignancy can masquerade as a common infectious process in a young, otherwise healthy individual, leading to a significant delay in diagnosis and treatment.

## Introduction

Primary mediastinal large B-cell lymphoma (PMBCL) is a distinct mature B-cell neoplasm that arises from thymic B-cells in the anterior mediastinum [1]. While historically categorized as a subtype of diffuse large B-cell lymphoma (DLBCL), it is now recognized as a unique clinicopathologic entity [1]. PMBCL accounts for approximately 2%-4% of all non-Hodgkin lymphomas and predominantly affects young adults, with a marked female preponderance [2]. The disease typically presents as a bulky mediastinal mass, often causing compressive symptoms such as cough, dyspnea, chest pain, and superior vena cava (SVC) syndrome, but initial manifestations may be non-specific [1-5]. The differential diagnosis for a large mediastinal mass is broad and requires a thorough workup of infectious and malignant etiologies.

Among infectious causes, actinomycosis is a rare consideration, as *Actinomyces* species can produce indolent, mass-forming lesions that mimic neoplastic processes. However, it is crucial to recognize that isolation of *Actinomyces* from sputum often represents oropharyngeal colonization rather than true invasive infection, which strictly requires histopathological confirmation of sulfur granules [6-8]. Conversely, PMBCL may be misdiagnosed as an infection if biopsy specimens reveal granulomatous inflammation. This overlap underscores the necessity for a multidisciplinary diagnostic approach [6-8].

Epidemiologically, PMBCL most commonly affects women in their third and fourth decades of life [1,9]. Most patients present with localized stage I-II disease, but the mass may invade adjacent thoracic structures, and pleural or pericardial effusions are common [3]. Systemic symptoms such as fever or weight loss are less frequent at presentation [9,10]. Despite the locally aggressive nature of the disease, the prognosis is excellent; modern immunochemotherapy regimens, such as DA-EPOCH-R (dose-adjusted etoposide, prednisone, vincristine, cyclophosphamide, doxorubicin, and rituximab), achieve long-term cure rates exceeding 85%-90% in young patients.

Pathophysiologically, PMBCL is characterized by dysregulation of the JAK-STAT and NF-κB signaling pathways, as well as immune evasion mechanisms including upregulation of PD-L1 and loss of B2M [1,2,5,10]. These molecular features have significant therapeutic implications, particularly regarding the utility of PD-1 inhibitors in relapsed disease. The tumor microenvironment is manipulated to suppress T-cell responses, further complicating host defense [4]. This case report describes a presentation of PMBCL initially confounded by an incidental *Actinomyces graevenitzii* finding, emphasizing the importance of integrating clinical, radiologic, and pathologic data to avoid diagnostic delay.

## Case presentation

**Figure 1:**
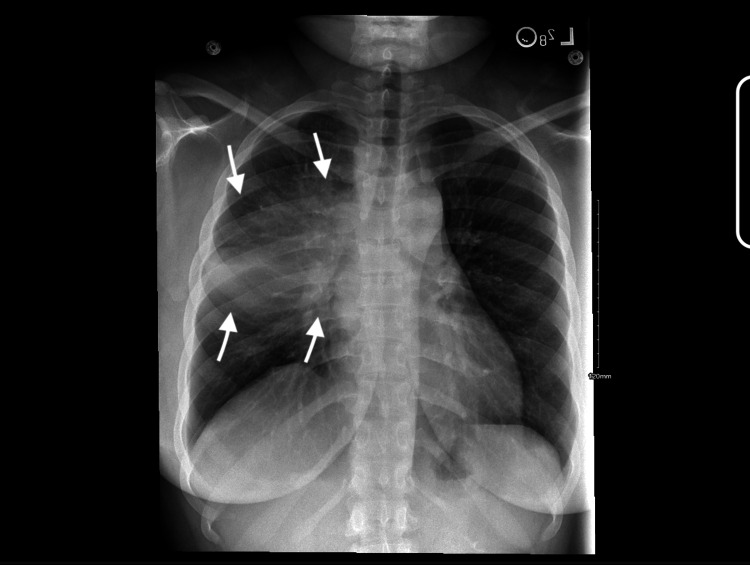
First chest X-ray performed during initial hospitalization prompting a workup for pneumonia

A 26-year-old African American female patient with a history of asthma presented to the Emergency Department (ED) at a large tertiary care hospital with a productive cough featuring blood-tinged sputum, shortness of breath, and new-onset facial swelling. Initial evaluation included a neck CT, which revealed findings concerning for cellulitis and prevertebral soft tissue swelling. She was diagnosed with right upper lobe pneumonia and discharged with Augmentin and azithromycin. Figure 1 depicts the initial chest X-ray obtained on admission.

Two days later, the patient saw her primary care provider (PCP) because her neck and facial swelling persisted. She was promptly referred to dental services, where a tooth extraction was performed to eliminate any potential odontogenic source for her facial symptoms.

Despite these interventions, the patient’s facial swelling did not resolve. She returned to the ED-now three weeks after her initial symptoms-with continued facial swelling, worsening cough productive of blood-tinged sputum, and new-onset night sweats. She denied fever, recent illness exposures, travel, or tuberculosis contact. Although she reported weight loss, she attributed this to intentional efforts (likely a retrospective constitutional "B" symptom). There was no smoking, alcohol, or IV drug use. Earlier the same day, her dentist confirmed there was no active dental infection.

**Figure 2:**
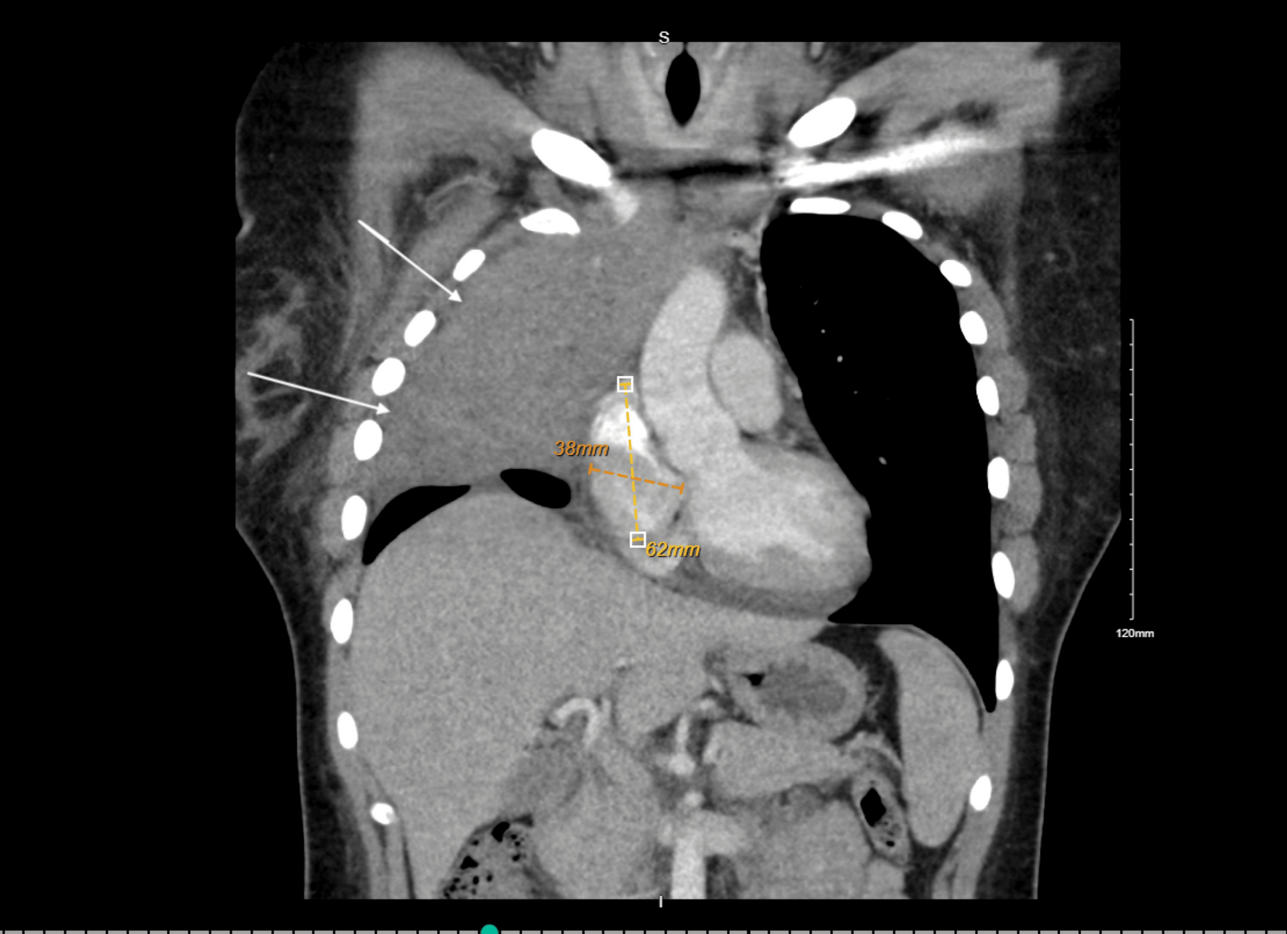
Well-defined soft tissue mass is noted within the mediastinum. The mass appears homogeneous in attenuation, with no obvious calcification, fat, or cystic components visible, with the arrows pointing toward the extension of the lymphoma

Further evaluations in the ED revealed significant leukocytosis (WBC 44,000/μL) with absolute neutrophilia, myelocyte precursors, monocytosis, anemia (hemoglobin 9.1 g/dL), and thrombocytosis (platelets 585,000/μL). Urinalysis was negative for infection. Chest CT performed at this stage demonstrated a right upper lobe mass with mediastinal involvement, regional lymphadenopathy, and a simple right-sided pleural effusion (Figure 2). She was admitted for workup of a suspected pulmonary neoplasm versus non-resolving pneumonia.

During admission, her WBC count progressively increased to 55,000/μL. Sputum culture grew *Actinomyces*; however, given the lack of specific abscess formation on imaging and the clinical context, this was interpreted as colonization. Infectious Disease initially recommended ceftriaxone, later escalating to Zosyn and vancomycin due to continued leukocytosis. Pulmonology recommended a biopsy. Table 1 outlines the findings of the workup done during the course of the stay, while Table 2 shows the flow cytometry findings. Fine-needle aspiration (FNA) from the right lung revealed medium to large atypical cells in a background of mixed inflammation, suspicious for lymphoma.

**Table 1:** Diagnostic tests performed

Test/analysis	Pertinent findings
Cytology	Atypical lymphoid cells present
Surgical pathology	Minute fragments of lung parenchyma with fibrosis; scattered medium to large atypical cells suspicious for lymphoma; atypical cells positive for CD20; negative for BCL1, BCL6, and CD10; BCL2 and CD23 equivocal; Ki-67 highlights a subset of cells
Fine-needle aspiration	Atypical lymphoid cells present; reactive pneumocytes present; no *Actinomyces* identified; rule out lymphoma
JAK2 mutation analysis	Negative
BCR/ABL1 kinase domain mutation	Negative
Flow cytometry	Large cells (3.6%): CD45+, CD19+, CD20+, CD5−, CD10−, CD23+, CD38+; small T lymphocytes (27%): CD4:CD8 ratio 4.2:1; small mature polytypic B lymphocytes (16%): kappa:lambda ratio 1.6:1; NK cells 0.56%; granulocytes 26%; remaining events represent uncharacterized cells and debris

**Table 2:** Summary of the current reference ranges with pertinent findings from the patient for the flow cytometry cell populations analyzed in this study

Cell population	Patient values	Reference ranges
Large cells	3.6%: CD45+, CD19+,CD20+, CD5−, CD10−, CD23+, CD38+	~1%–5% of lymphocytes
Small T lymphocytes (total)	27%	~60%–85% of lymphocytes
CD4:CD8 ratio	4.2:1	1.0–4.0:1
Small mature B lymphocytes	16%; polytypic	~5%–20% of lymphocytes
Kappa:lambda ratio	1.6:1	0.26–1.65:1
NK cells	0.56%	~5%–20% of lymphocytes
Granulocytes	26%	~50%–70% of total nucleated cells
Uncharacterized cells	Remaining cells in the field	Minimal to none
Debris	Remaining cells in the field	Minimal to none

Initial vitals in the ED are as follows: blood pressure (BP) 128/82, heart rate (HR) 118, respiration rate (RR) 19, temp 98.4°F, SpO_2_ 98% on room air, weight 76.2 kg, and pain score 4. Noted comorbidities included hypoalbuminemia, metabolic acidosis, and acute kidney injury (AKI), likely secondary to hypoperfusion or nephrotoxic exposure.

## Discussion

This case illustrates a striking diagnostic dilemma in a young woman whose PMBCL presented as a convincing mimic of an infectious disease. The initial constellation of symptoms-including facial swelling, cough, fatigue, and imaging suggestive of a right upper lobe pneumonia with associated soft tissue inflammation-reasonably directed the initial workup toward an infectious etiology. This clinical scenario underscores the profound risk of cognitive errors in medicine, particularly anchoring bias, where an early plausible diagnosis can prevent the consideration of alternative, more serious possibilities, even as contradictory evidence emerges. The failure of the patient to improve as expected with standard therapy was a critical turning point that necessitated a re-evaluation of the entire clinical picture [11-13].

The evolving differential diagnosis: from infection to malignancy

The initial working diagnosis of community-acquired pneumonia was logical, prompting appropriate treatment with broad-spectrum antibiotics. However, several features deviated significantly from a typical infectious course. The most prominent red flag was the patient’s facial swelling, which was most pronounced upon waking. This is a classic hallmark of SVC syndrome, a clinical emergency resulting from the extrinsic compression of the SVC. This compression elevates venous pressure in the head, neck, and upper extremities, leading to edema that is exacerbated by the recumbent position during sleep. While rare infectious processes can cause this, a rapidly growing anterior mediastinal neoplasm is the most common cause in young adults, and its presence should have immediately prompted an investigation for malignancy [12,14,15].

The diagnostic picture was complicated by the isolation of *Actinomyces *from sputum. However, *Actinomyces *is a common commensal of the oropharynx, and sputum positivity in the absence of sulfur granules or tissue invasion often reflects colonization, particularly following dental procedures [16,17]. True thoracic actinomycosis typically progresses slowly, whereas this patient's symptoms worsened dramatically over weeks.

Ultimately, the failure to respond to antibiotics and the rising leukocytosis (peaking at 55,000 cells/µL) were key pivots. This profound neutrophilia represents a leukemoid reaction, a paraneoplastic phenomenon often mediated by tumor secretion of granulocyte colony-stimulating factor (G-CSF) [16]. This solidified the suspicion for malignancy, placing PMBCL high on the differential alongside thymoma, germ cell tumors, and thyroid neoplasms [11-13].

Pathophysiology and definitive diagnosis of PMBCL

PMBCL is a distinct, aggressive B-cell lymphoma that accounts for 2%-4% of all non-Hodgkin lymphomas and demonstrates a striking predilection for young women [12,13]. Pathophysiologically, it arises from thymic B lymphocytes in the mediastinum. Its aggressive behavior is driven by characteristic genetic abnormalities, including the dysregulation of the JAK-STAT and NF-κB signaling pathways, which promote cell survival and proliferation. Furthermore, amplification of the 9p24.1 locus leads to the overexpression of immune checkpoint ligands PD-L1 and PD-L2, allowing the tumor to evade the host's immune surveillance [18,19]. These pathways are targets for emerging therapies, such as checkpoint inhibitors (e.g., pembrolizumab) in relapsed cases. The tumor often incites a desmoplastic reaction, leading to extensive sclerosis or fibrosis, which makes obtaining a diagnostic sample via FNA difficult, often yielding scant or inconclusive results, as was initially seen in this patient.

The patient’s entire symptom constellation can be explained by the tumor's pathophysiology. The local compressive effects of the bulky mediastinal mass led directly to SVC syndrome (facial swelling), tracheal compression (cough), and esophageal compression ("throat swelling"). The systemic symptoms of fatigue, drenching night sweats, and weight loss are classic constitutional “B symptoms” of lymphoma, driven by a massive release of inflammatory cytokines from the tumor that creates a high metabolic and catabolic state [11,12]. Likewise, the leukemoid reaction is attributed to the paraneoplastic secretion of hematopoietic growth factors, such as G-CSF, by the tumor cells [12,20].

A definitive diagnosis was achieved with an endobronchial ultrasound (EBUS)-guided biopsy, which provided adequate tissue for a full histopathological and immunophenotypic analysis. The characteristic profile of tumor cells positive for the B-cell marker CD20 but negative for CD5 and CD10, combined with a high Ki-67 proliferative index, confirmed the diagnosis of PMBCL [12,18].

It is pertinent to note that the path to diagnosis required progressively more invasive procedures, moving from a limited FNA to an EBUS-guided biopsy that provided adequate tissue. The immunohistochemical profile was essential for the definitive diagnosis. The tumor cells were positive for CD20, a pan-B-cell marker, confirming their lineage. Crucially, they were negative for CD5 and CD10. This profile helps distinguish PMBCL from other lymphomas; for instance, a subset of DLBCL is CD10-positive, and small lymphocytic lymphoma/chronic lymphocytic leukemia is typically CD5-positive. The high Ki-67 proliferative index highlighted the aggressive, rapidly dividing nature of the cancer, aligning with the patient's rapid clinical progression. This complete immunophenotype, in the context of a large anterior mediastinal mass in a young female patient, is classic for PMBCL.

Finally, the management plan appropriately addressed two key facets of care for this patient population. The initiation of high-dose prednisone serves as a bridging therapy to reduce tumor burden and alleviate compressive symptoms while awaiting definitive treatment. Critically, the immediate referral for an oncofertility consultation is the standard of care. The chemotherapeutic regimens used to treat PMBCL are highly effective but carry a significant risk of causing premature ovarian failure and infertility, making fertility preservation a crucial discussion before treatment begins.

Management considerations

Standard first-line therapy for PMBCL is DA-EPOCH-R, which has demonstrated superior outcomes compared to R-CHOP (rituximab, cyclophosphamide, hydroxydaunorubicin (doxorubicin), oncovin (vincristine), and prednisone), often eliminating the need for consolidative radiation. Given the high risk of premature ovarian failure with these regimens, immediate referral for oncofertility consultation is the standard of care [11,13,18,19]. This case powerfully demonstrates that a young patient's presentation of persistent, atypical "infectious" symptoms, particularly when accompanied by signs of mediastinal compression and extreme laboratory abnormalities, must prompt an aggressive and timely investigation for an underlying malignancy.

## Conclusions

This case of a 26-year-old woman with PMBCL vividly demonstrates the challenge of diagnosing this aggressive malignancy when it masquerades as a common infection. The patient’s initial presentation created a compelling picture of pneumonia, which was later complicated by the confounding discovery of *Actinomyces*, a finding that could have further anchored the diagnosis to an infectious etiology.

However, several critical findings were inconsistent with this narrative: the presence of clinical signs was initially indicative of pneumonia followed by *Actinomyces *infection. The presence of clinical signs indicative of SVC syndrome, a failure to respond to robust antimicrobial therapy, and a profound paraneoplastic leukemoid reaction were red flags that correctly shifted the investigation toward an underlying malignancy. This case highlights the dangers of anchoring bias and the absolute necessity of maintaining a broad differential diagnosis, even in a young and otherwise healthy patient. When a patient's clinical trajectory deviates sharply from the expected course of a common illness, malignancy must be aggressively pursued to prevent critical delays in diagnosis and treatment. Ultimately, this case underscores the importance of vigilance and reinforces the essential role of prompt, multidisciplinary management, including crucial oncofertility consultation, to optimize outcomes and preserve the future quality of life for young adults diagnosed with cancer.
